# A Novel Nonvascular Application of the Steerable Microcatheter

**DOI:** 10.7759/cureus.3469

**Published:** 2018-10-19

**Authors:** Erik Eadie, Taylor S Harmon, Erik Soule, Paul C Hulsberg, Michael Shabandi, Jerry Matteo

**Affiliations:** 1 Interventional Radiology, University of Florida College of Medicine, Jacksonville, USA; 2 Interventional Radiology, The University of Texas Medical Branch, Galveston, USA

**Keywords:** staghorn calculus, percutaneous nephrostomy, steerable microcatheter, nephro-ureteral stent

## Abstract

The percutaneous nephrostomy (PCN) is a relatively common interventional procedure used to treat a multitude of nephro-urological conditions. Traditionally, interventional radiologists use ultrasound guidance, needles, catheters, and guidewires to access the collecting system percutaneously. The placement of a nephro-ureterostomy stent may be precluded by challenging renal calyx anatomy or an underlying disease process that obstructs placement. In cases of complex obstruction, accessing the renal collecting system may require deviation from conventional methods. We present a case that after many failed attempts with a wide variety of guidewires and catheters, a steerable microcatheter (SMC) was used to safely and effectively access the renal collecting system. This novel technique utilizes the SMC to efficiently achieve complicated PCN stent placement, relieving the renal drainage system obstruction and potentially minimizing or avoiding complications, such as urosepsis and/or renal failure.

## Introduction

Percutaneous nephrostomy (PCN) was first described in 1955 as a novel technique by interventionist Goodwin et al. for the relief of hydronephrosis [1]. Later in 1976, PCN was adapted for relieving calculus obstructions under radiological guidance in the first ever percutaneous lithotomy [2]. Since these procedural innovations, PCN and subsequent stent placement have been the standard of care for renal pathologies affecting the pyelocaliceal system. Interventionists gain access to the upper urinary tract renal collecting system in an antegrade fashion in order to perform different diagnostic and therapeutic procedures [3]. PCN has now been applied diversely in other clinical settings; most recently, it is the standard of care to perform PCN in patients requiring a urinary diversion, including but not limited to obstructive neoplasia, pyelonephritis, or pathologic fistula formation [4-5].  

In 1997, the first percutaneous “tubeless” lithotomy was performed, challenging the clinical use of interventional PCN placement prior to lithotomy [6]. Rather than accessing ureteral calculi via PCN and subsequent lithotomy, the tubeless surgical procedure entails the retrograde placement of a double J stent through the urethra, bladder, and ureters, allowing the removal of the identified calculus [6]. However, in recent years since the development of this surgical method, the literature shows that there are many complications associated with tubeless lithotomy, including but not limited to hemorrhage, extravasation, or perforation of the genitourinary parenchyma [7]. Furthermore, surgical tubeless lithotomy has a highly associated infection risk in patients receiving this surgical procedure, resulting in the necessity of PCN stent placement regardless of the method of lithotomy performed [8]. With the current surgical and interventional literature available, the paradigm for the management of renal and ureteral calculi must include the placement of PCN stents.

As the indication for interventional PCN has evolved and practicality broadened to encompass many uses since its conception, the current devices used for procedural success in these cases are sometimes limiting to the operator. Particularly, complex renal disease may challenge the operator, such that the available guidewires and catheters used to access the renal parenchyma, ureters, and urinary bladder fail to traverse any associated anatomical renal obstruction. Large staghorn calculi or struvite stones, for example, can become so obstructive that conventional guidewires and catheters are unable to gain urinary intraluminal access. However, the development of the steerable microcatheter (SMC) offers a solution for complex obstructive renal disease, such as large struvite stone formation. The literature has only documented one technical report of an SMC used to increase intravascular procedural efficiency [9], but there have been no reports of similar use in the extravascular setting. The following case presented will demonstrate the advantageous use of an SMC for PCN stent placement complicated by large staghorn calculus compared to the conventional use of typical catheters and guidewires.

## Case presentation

**Figure 1:**
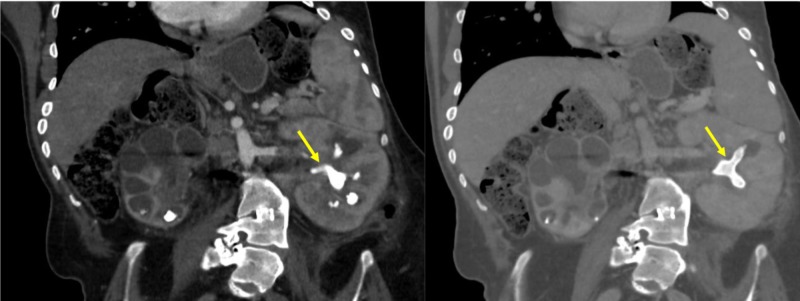
The left and right contrast-enhanced coronal computed tomography (CT) images show a large staghorn calculus in the left kidney (yellow arrow)

**Figure 2:**
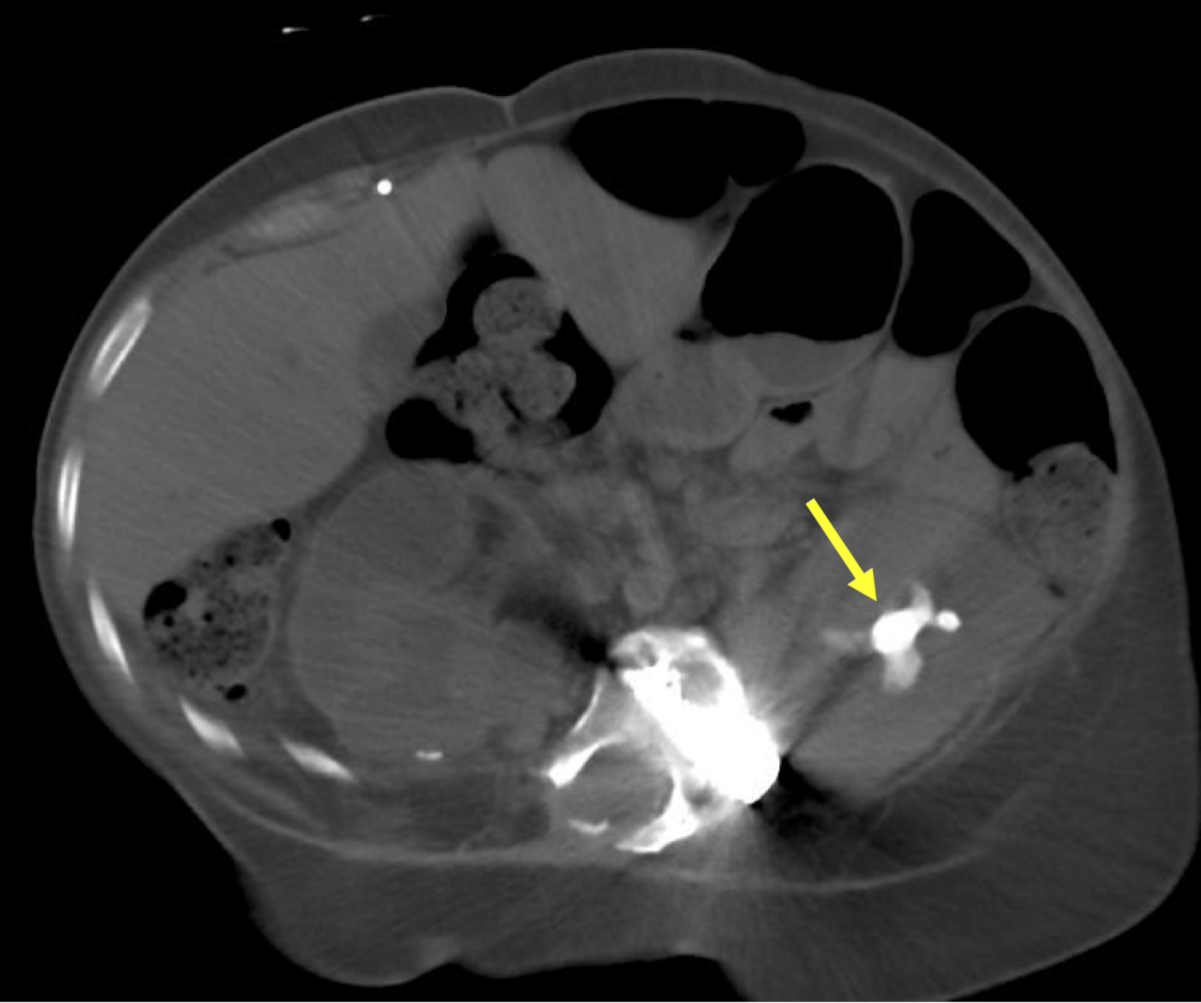
A non-contrast axial computed tomography image showing a large staghorn calculus (yellow arrow)

**Figure 3:**
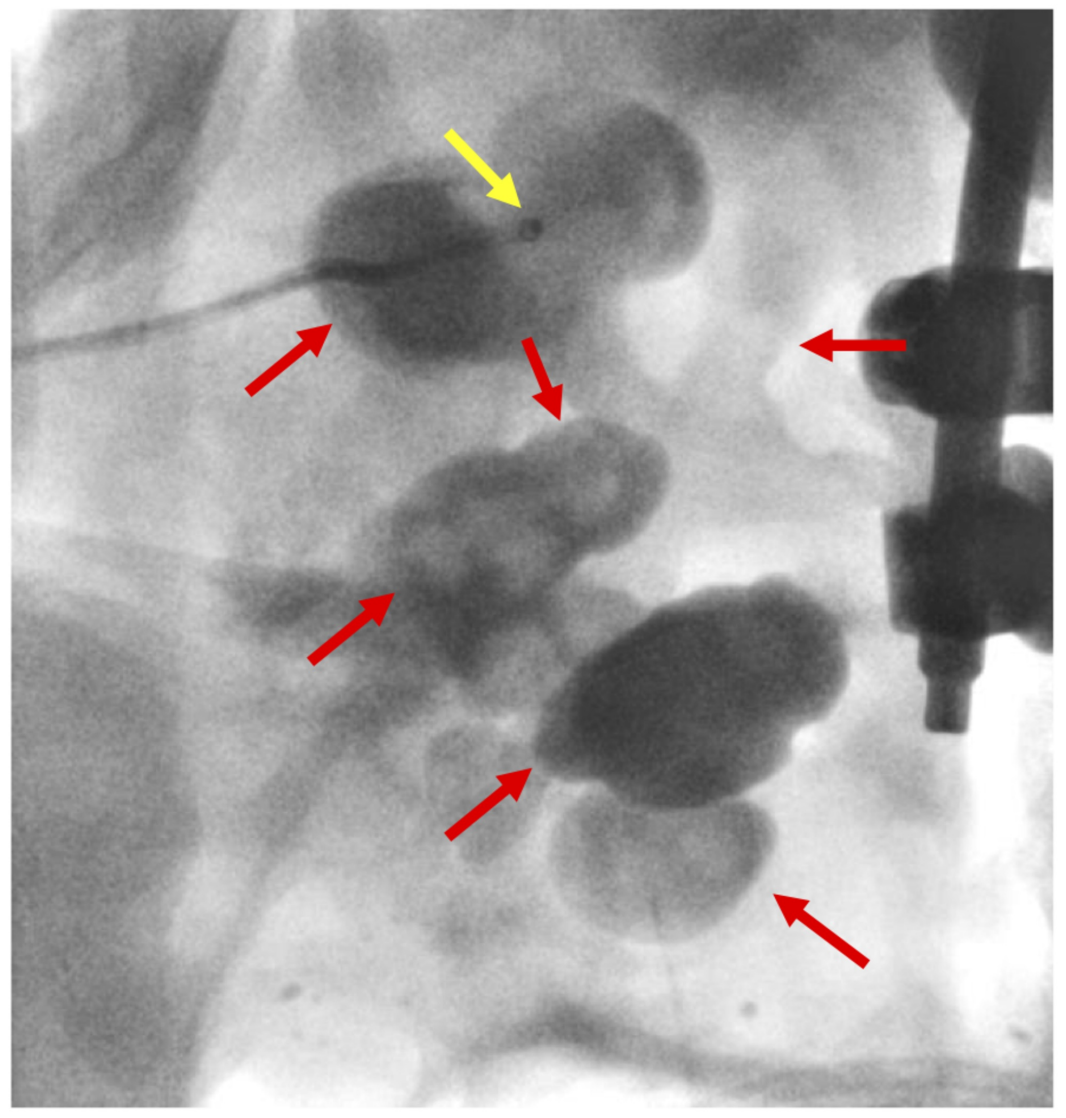
A fluoroscopic image of the left kidney is shown at the access site The yellow arrow indicates the tip of the base catheter within the superior calyx. The red arrows indicate the stone cast and extensive staghorn calculus.

A 28-year-old female presented with a staghorn calculus (Figures 1-2) and in need of a nephro-ureterostomy stent placement. Using fluoroscopic guidance, a 21-gauge needle was introduced through the skin into an inferior renal calyx and an antegrade pyelogram was performed demonstrating opacification of the collecting system. After demonstrating return of urine, a wire was passed through the needle and a dilator was introduced over the wire. The existing wire was removed and multiple catheters and wires were subsequently used to attempt to gain access to the collecting system. After unsuccessful attempts through the inferior calyx, the superior renal calyx was attempted in the same manner. Due to the obstructive staghorn calculus, this was also unsuccessful (Figure 3).

**Figure 4:**
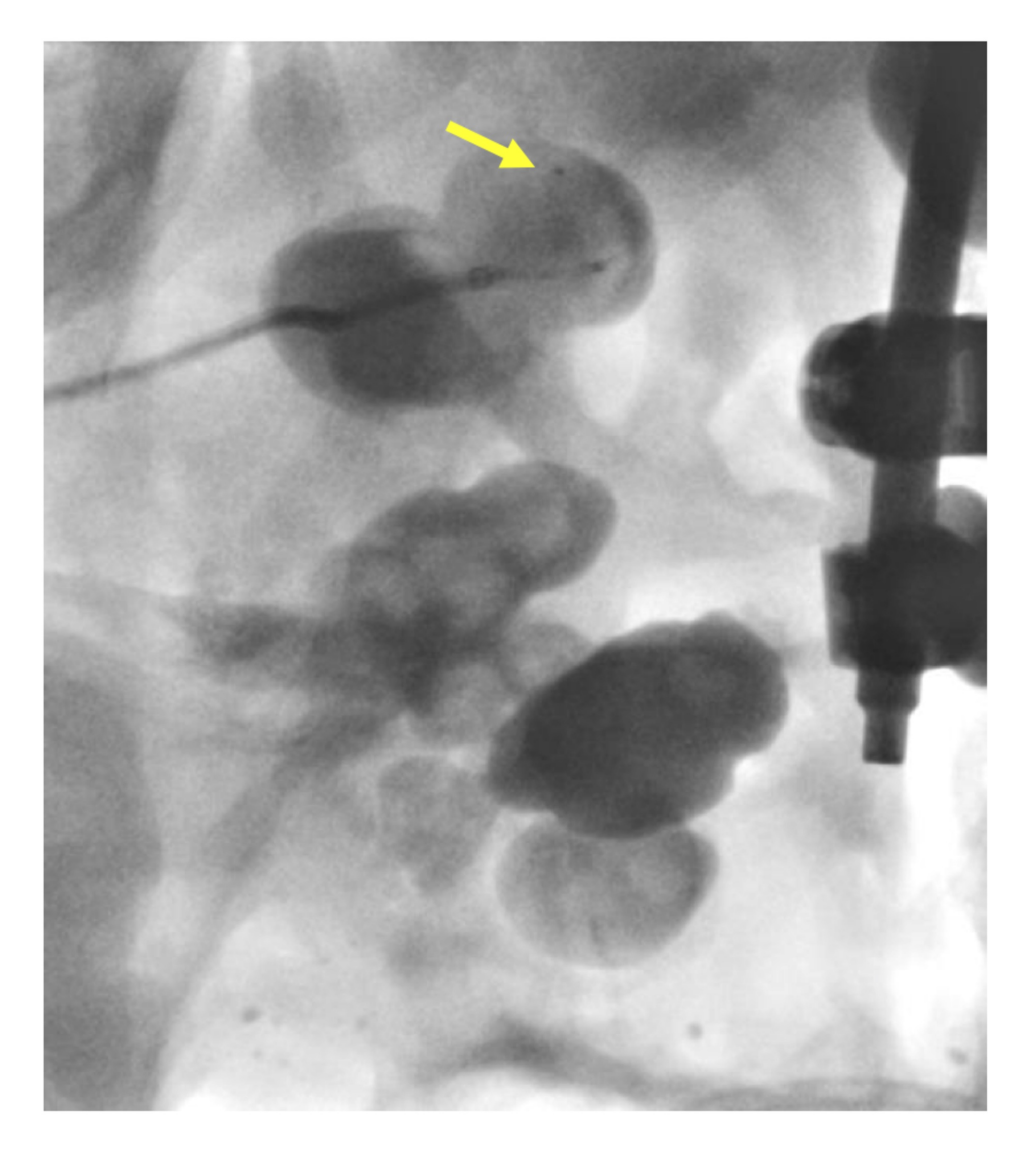
Insertion of the steerable microcatheter (yellow arrow) into the renal calyx

**Figure 5:**
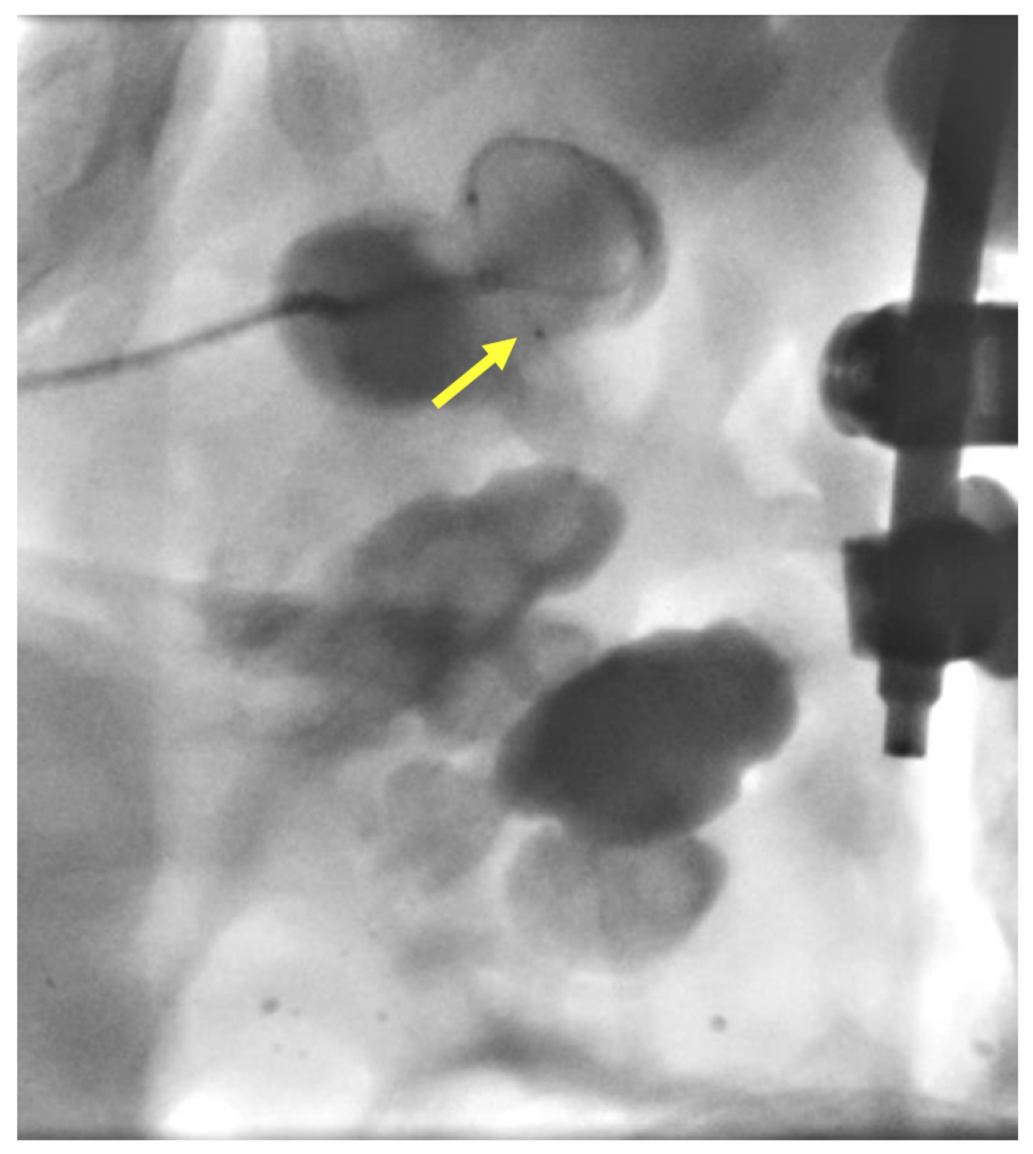
Navigation of the steerable microcatheter (yellow arrow) within the renal calyx and around the staghorn calculus

**Figure 6:**
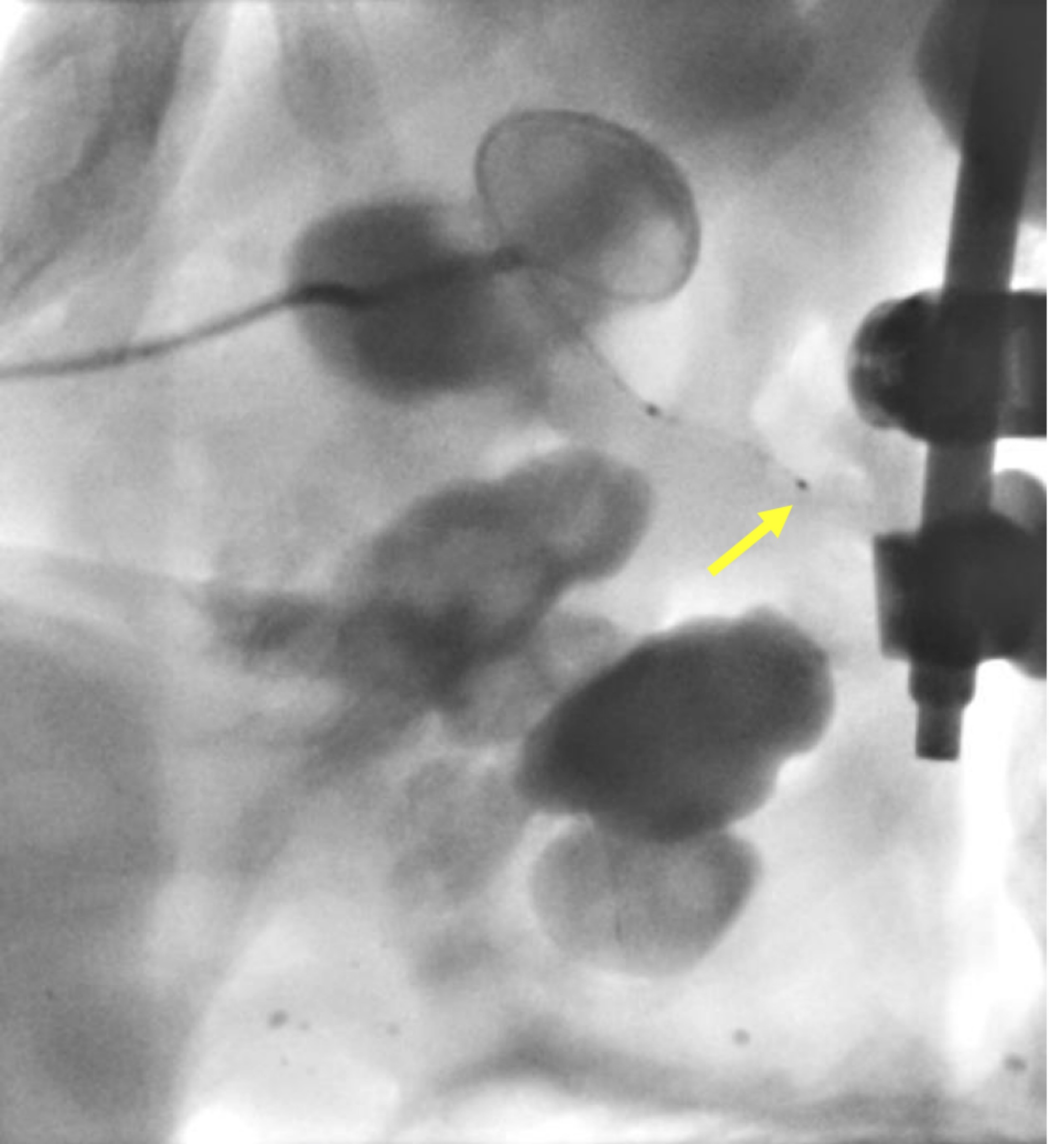
Advancement of the steerable microcatheter (yellow arrow) around the staghorn calculus and into the renal pelvis.

**Figure 7:**
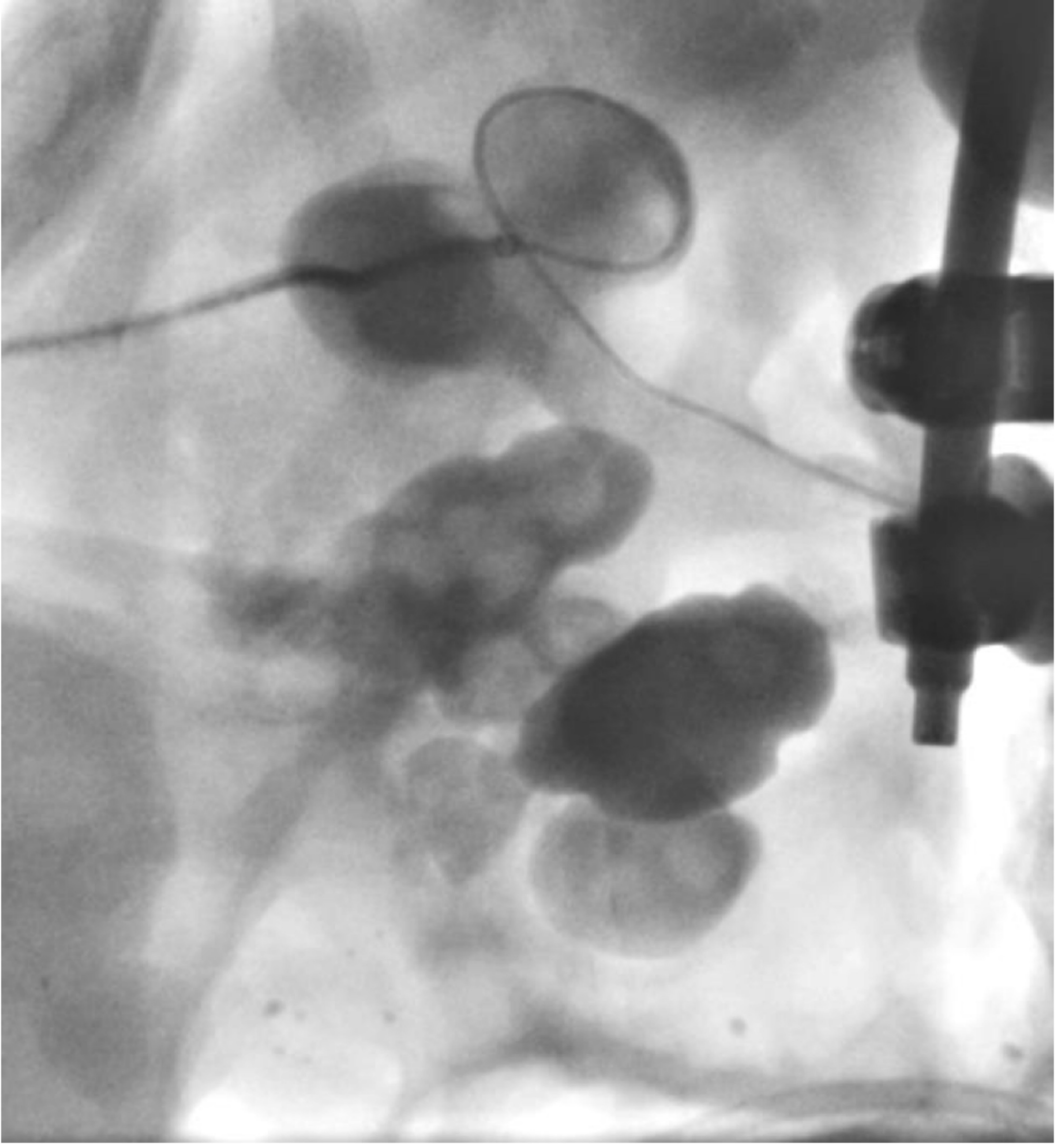
Further advancement of the steerable microcatheter around the staghorn calculus into the proximal ureter

After approximately two hours of procedure time and several unsuccessful attempts to access the central renal pelvis, it was evident the procedure might have to be abandoned. As a final attempt, the decision was made to use a SwiftNINJA® SMC (Merit Medical Systems, South Jordan, UT). This catheter easily circumnavigated around the staghorn calculus through the left renal collecting system and eventually into the proximal ureter (Figures 4-7). This maneuver was completed within one minute.  

**Figure 8:**
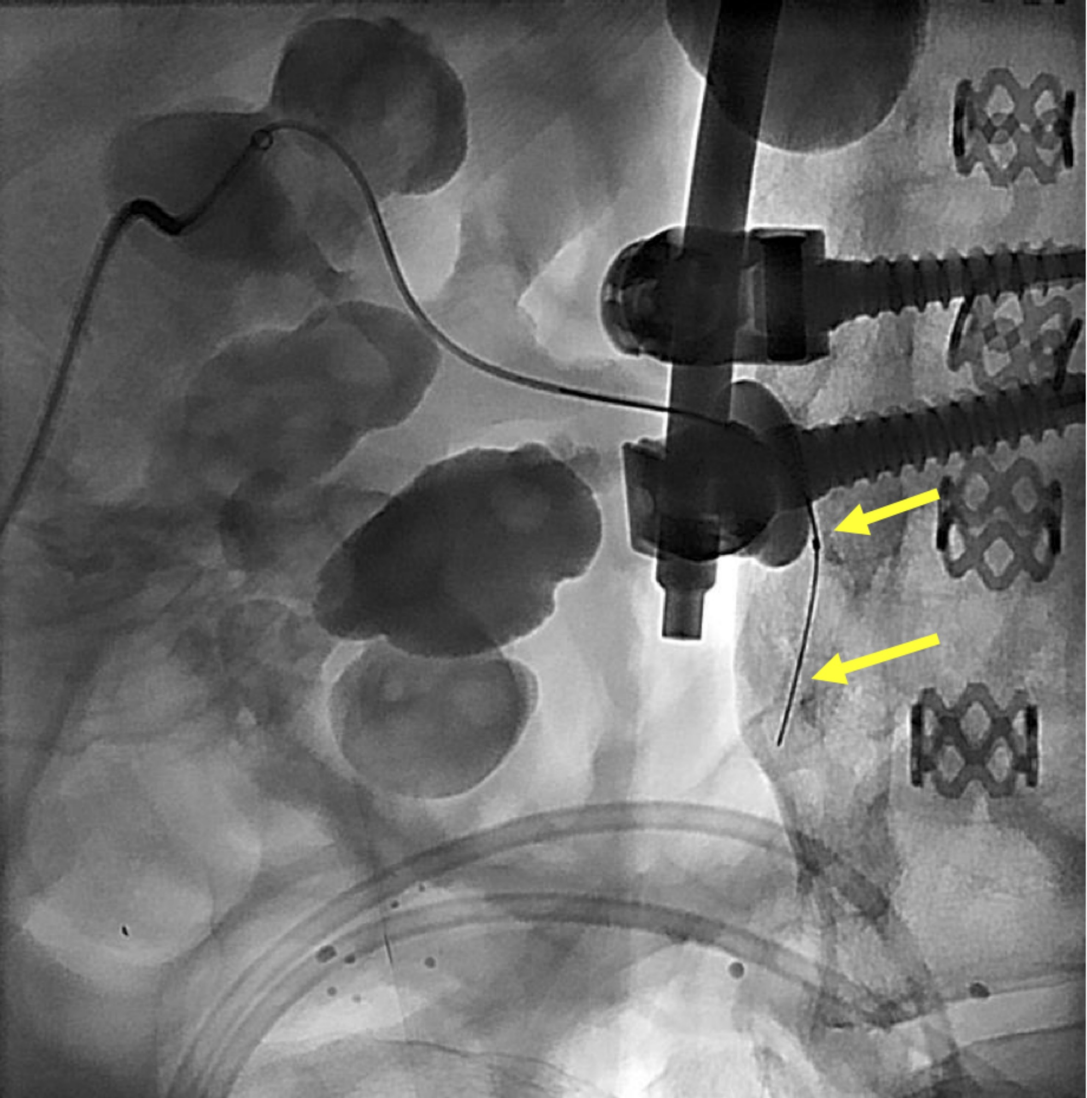
The steerable microcatheter is shown successfully traversing the renal collecting system with tip in the ureter The top yellow arrow shows the tip of the steerable microcatheter; the bottom yellow arrow shows the tip of the guidewire.

**Figure 9:**
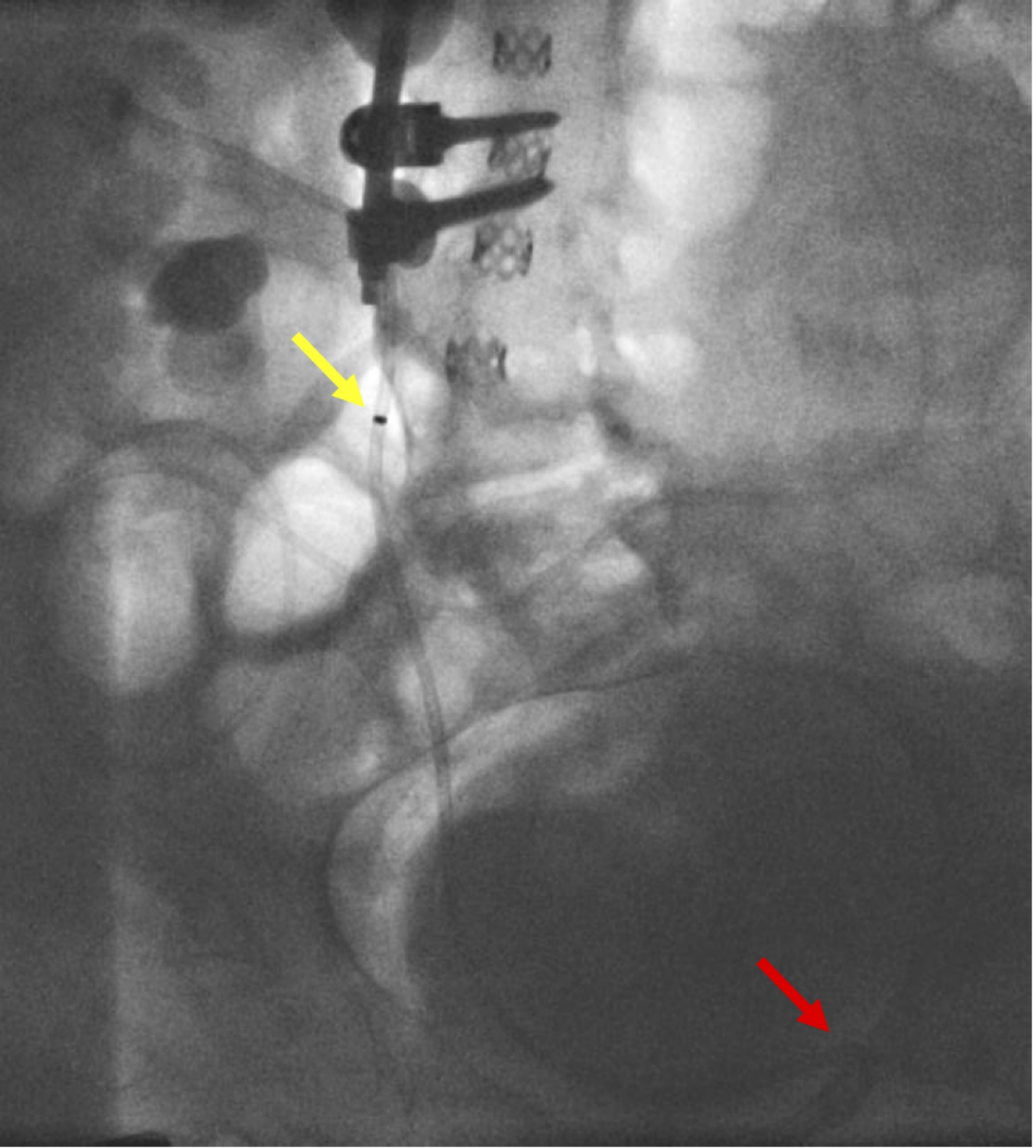
Placement of the nephro-ureterostomy stent The yellow arrow indicates the proximal radiopaque marker of the catheter. The red arrow demonstrates the distal end of the catheter forming a pigtail in the urinary bladder. The proximal pigtail was unable to be formed due to the space-occupying staghorn calculi within the renal pelvis.

**Figure 10:**
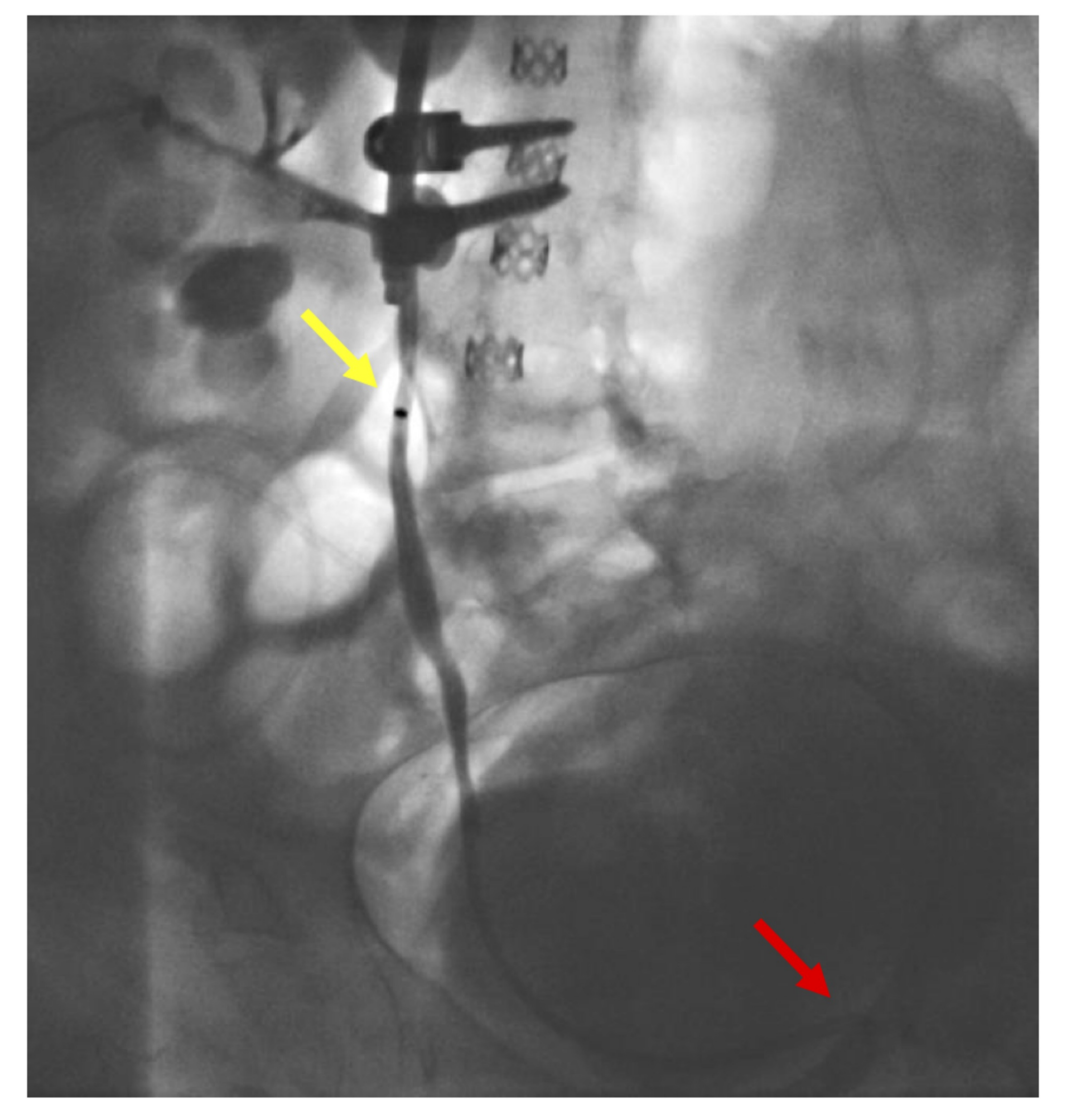
A contrast injection is shown to confirm nephroureterostomy stent placement The yellow arrow indicates the proximal radiopaque marker of the catheter. The red arrow demonstrates the distal end of the catheter.

A Platinum Plus™ guidewire (Boston Scientific, Marlborough, MA) was used to guide the SMC into the bladder (Figure 8). The SMC was then exchanged for a 4-French Berenstein catheter, which was then exchanged over a stiff guidewire for an 8.5-French nephro-ureterostomy stent (Cook Medical, Bloomington, IN). The distal loop of the stent was formed in the urinary bladder (Figure 9). Due to the space-occupying staghorn calculus, the proximal loop was unable to be formed in the renal pelvis. Contrast was injected through the stent demonstrating flow into the urinary bladder (Figure 10). Hemostasis was achieved and the nephro-ureterostomy stent was secured in place with a fixation device and capped.

## Discussion

Due to the length of the procedure and the indwelling infection caused by the staghorn calculus, the patient developed hypotension and septic shock requiring vasopressors and admission to the intensive care unit. This highlights the emergent nature of PCN in the obstructed, infected kidney. Complex renal and ureteral calculi can delay PCN access, even for highly skilled interventionists. PCN stent placement is limited to anatomic calculus obstruction, the fluoroscopic field of view, and the limitations inherent to guidewires and catheters. Specifically, staghorn or struvite calculi offer the most onerous challenge when attempting to place PCN stents since they tend to be large and form to the shape of the renal calyces, forming a cast. In these cases, the conventional passage for guidewires and catheters distal to struvite stones is hindered from advancement into the ureters and urinary bladder.

With the development of steerable microcatheters (SMCs), the maneuverability through complex obstructions or tortuosities is efficiently manageable [9]. Though the literature is currently limited to only one documented account of intravascular interventional use of a wireless steerable microcatheter [9], the presented case described applied the same technique in the extravascular setting. Specifically, it has shown that an SMC can be used when PCN fails with the conventional use of a needle, guidewire, and traditional catheter access in a patient with a large obstructing staghorn calculus.

Though less common, open urological surgery is the standard of care for a select patient population with complex renal disease. In the reported literature, the most common indications for open renal lithotomy are a complex stone burden, failure of extracorporeal shock wave lithotripsy or endourological treatment, genitourinary abnormalities, morbid obesity, and co-morbid medical disease [10]. In the case of a majority or completely obstructive staghorn calculus, such as that presented in this report, interventional PCN and lithotomy may be precluded and open surgery may be indicated. Using a novel extravascular application for the SMC, a successful resolution of the increased renal pressure was achieved. Failed PCN may be averted using the SMC for complex obstructive renal disease, obviating the need for surgery.

With the pressure to drive down healthcare costs, some might say that right now the SMC is too expensive for use in a PCN. What should be taken into consideration is the time that using a device to make the procedure faster and more efficient could save. In this case, if we had initially used the SMC, then we could have saved hours of time in the fluoroscopy suite. The time spent on any given case can have an exponential financial impact due to staffing, time away from other procedures, and supplies used (i.e., standard catheters and wires). The case time is also directly related to radiation exposure to the patient. As with any fluoroscopic procedure, we should decrease radiation exposure to patients. Thus, the faster and more efficient PCN placement with the SMC will have significantly less radiation exposure to the patient.

## Conclusions

The innovative history of the interventional PCN has led to management advancements in patients with nephro-urological pathologies. The paradigm is forever shifting toward less invasive treatment modalities for these diseases as devices have become increasingly practical in function. In the case of patients with complex obstructive renal disease as a result of large staghorn calculi, SMCs may be indicated due to increased functionality over the traditional use of guidewires and catheters, especially when there is a need to obtain rapid PCN access which could lead to better patient outcomes. This is the first reported extravascular use for the SMC, a testimony to innovative applications of devices that may improve patient outcomes.
